# Supplementary material for: Pregnancy Apps for Self-Monitoring: Scoping Review of the Most Popular Global Apps Available in Australia
Source: Int J Environ Res Public Health. 2023 Jan 5;20(2):1012. doi: 10.3390/ijerph20021012 (PMC9858738; doi:10.3390/ijerph20021012)
Supplement: Supplementary file 1 [file ijerph-20-01012-s001.zip › ijerph-2088281-supplementary/Supplementary_Material_appreview_4.01.23/Supplementary_CodeS1_Appstorescrapers.pdf]

Supplementary material – javascript code used for app store scraping

Code used to search main app stores

Apple App Store scraper:

```
//Code modified from https://github.com/facundoolano/app-store-scraper
//These are the dependencies, you need 'converter' to convert the output into a csv
//fs syncs the csv to file
//gplay is our main function to scrape Apple App Store (iOs devices)! Yay!

const converter = require('json-2-csv');
const fs = require('fs')
var store = require('app-store-scraper');

//You then want to define what the search terms are and you can search for multiple at once (do not list too
many, just run it again as it can lag)
//if for some reason it does not run with more than one, just run it one at a time :)

let listOfSeachTerms = [
    "pregnancy",
    "pregnancy"
];

//This is just saying grab i number of of search terms from the list
for(var i = 0; i < listOfSeachTerms.length; i++){
    getAppDetails(listOfSeachTerms[i])
}

//Defining the scrape parameters: by default, the number of apps that it will scrape is the 250 most popular apps
in response to that search term
//You can add parameters for country and language, I had added an example below
async function getAppDetails(searchTerm) {
    let res = await store.search({
        throttle: 5,
        term: searchTerm,
        num: 200,
        lang: 'lang',
        country: 'au',
        page: 1,
        fullDetail: true
    })
    // res = await getFullRes(res); // res is now an array of json objects with full details of each app
    console.log(res);

    converter.json2csv(res, (err, csv) => {
        if (err) {
            throw err;
        }

        // print CSV string
        // console.log(csv);
```

```
        // write CSV to a file
        fs.writeFileSync(`${searchTerm}.csv`, csv);
    });
}
```

Google Play Store scraper:

```
//Code modified from https://github.com/facundoolano/google-play-scraper
//These are the dependencies, you need 'converter' to convert the output into a csv
//fs syncs the csv to file
//gplay is our main function to scrape google playstore (android devices)! Yay!
const converter = require('json-2-csv');
const fs = require('fs')
var gplay = require('google-play-scraper');

//You then want to define what the search terms are and you can search for multiple at once (do not list too
many, just run it again as it can lag)
//if for some reason it does not run with more than one, just run it one at a time :)
let listOfSeachTerms = [
    "pregnancy"
];

//This is just saying grab i number of of search terms from the list
for(var i = 0; i < listOfSeachTerms.length; i++){
    getAppDetails(listOfSeachTerms[i])
}

//Defining the scrape parameters: by default, the number of apps that it will scrape is the 250 most popular apps
in response to that search term
//You can add parameters for country and language, I had added an example below
async function getAppDetails(searchTerm) {
    let res = await gplay.search({
        throttle: 5,
        term: searchTerm,
        num: 100,
        lang: 'eng',
        country: 'au',
        fullDetail: true
    })
    // res = await getFullRes(res); // res is now an array of json objects with full details of each app
    // You will get a nice set of app details from this array!
    console.log(res);

    //Now time to convert the json array to a csv
    converter.json2csv(res, (err, csv) => {
        if (err) {
            throw err;
        }

        // print CSV string
        // console.log(csv);
```

```
        // write CSV to a file
        //csv name will be the searchterm so for search term pregnancy, it will be called pregnancy.csv
        fs.writeFileSync(`${searchTerm}.csv`, csv);
    });
}
```

Code used to search app store categories

Apple App Store category scraper:

```
//These are the dependencies, you need 'converter' to convert the output into a csv
//fs syncs the csv to file
//gplay is our main function to scrape apple app store categories (iOs devices)! Yay!

const converter = require('json-2-csv');
const fs = require('fs')
var store = require('app-store-scraper');

//List the categories you want to search
let listOfSeachTerms = [
    store.category.HEALTH_AND_FITNESS
];

for(var i = 0; i < listOfSeachTerms.length; i++){
    getAppDetails(listOfSeachTerms[i])
}

//The searchterms retrieved from each of these categories
async function getAppDetails(title) {
    let res = await store.list({
        category: title,
        collection: store.collection.TOP_FREE,
        num: 200,
        lang: 'en',
        FullDetail: true
    });
    console.log(res);

    converter.json2csv(res, (err, csv) => {
        if (err) {
            throw err;
        }

        // print CSV string
        // console.log(csv);

        // write CSV to a file
        fs.writeFileSync( `${title}.csv`, csv);
    });
}
```

Google Play Store category scraper:

```
//These are the dependencies, you need 'converter' to convert the output into a csv
//fs syncs the csv to file
//gplay is our main function to scrape google playstore categories (android devices)! Yay!

const converter = require('json-2-csv');
const fs = require('fs')
var gplay = require('google-play-scraper');

//The searchterms retrieved from each of these categories
let listOfSeachTerms = [
    gplay.category.MEDICAL,
    gplay.category.EDUCATION,
    gplay.category.LIFESTYLE,
    gplay.category.PARENTING,
    gplay.category.HEALTH_AND_FITNESS,
];

for(var i = 0; i < listOfSeachTerms.length; i++){
    getAppDetails(listOfSeachTerms[i])
}

//The top free apps in each category
async function getAppDetails(title) {
    let res = await gplay.list({
        category: title,
        collection: gplay.collection.TOP_FREE,
        num: 500,
        lang: 'en',
        FullDetail: true
    });
    console.log(res);

    converter.json2csv(res, (err, csv) => {
        if (err) {
            throw err;
        }

        // print CSV string
        // console.log(csv);

        // write CSV to a file
        fs.writeFileSync( `${title}.csv`, csv);
    });
}
```
