# Supplementary material for: Pregnancy Apps for Self-Monitoring: Scoping Review of the Most Popular Global Apps Available in Australia
Source: Int J Environ Res Public Health. 2023 Jan 5;20(2):1012. doi: 10.3390/ijerph20021012 (PMC9858738; doi:10.3390/ijerph20021012)
Supplement: Supplementary file 1 [file ijerph-20-01012-s001.zip › ijerph-2088281-supplementary/Supplementary_Material_appreview_4.01.23/Supplementary_TableS1_AppScoringCriteria.pdf]

Supplementary Tables – App scoring criteria

SCORECARD Approach – rating app quality [44]. Newly added criterion is in red font.

Technical Domain

Domain	Title	Assessment Criteria	Points (0=none; 1 = partial; 2 = full)	Section Total
Technical	Security	Is all data encrypted (both in flight and at rest)?<br>Flight = when data is being moved to a cloud storage or being shared via email/message. At rest = data stored within the app or on the phone.	0 to 2<br>(If the app does not mention data encryption or it is not obvious= 0)	Add up individual scores
Technical	Security	Does the application require a login?	0 to 2	
Technical	Security	Does the application support 3rd party identity providers (e.g. Google, Facebook, etc.)	0 to 2	
Technical	Security	Does the app provide a logout mechanism?	0 to 2	
Technical	Security	Does the application send sensitive data via e-mail?	0 to 2	
Technical	Security	Does the application include features of strong authentication such as password strength, security questions, and tokens?	0 to 2	
Technical	Privacy	Does the application require necessary access to phone components (e.g. Camera, Microphone, etc.)?	0 to 2	Add up individual scores
Technical	Privacy	Does the application have a data use agreement / terms of service?	0 to 2	
Technical	Privacy	Does the application request consent for use of data?	0 to 2	
Technical	Privacy	Does the application give the user the ability to control what data is shared?	0 to 2	
Technical	Privacy	Does the application have a privacy policy?	0 to 2	
Technical	Privacy	Does the application state compliance with HIPAA (1) and/or GDPR (2)	0 to 2	
Technical	Privacy	Data sharing w/ third parties- The following data is NOT used to track you across apps and website owned by other companies and linked to your identity (contact info, contacts, usage data,user content, location, identifiers, financial info, sensitive	0 to 1	

		info, search history, diagnostics, purchases, health and fitness, other data)?		
Technical	Interoperability	Does the application provide a means to export user's data (e.g., PDF (1), CSV (2), JSON (2))?	0 to 2	Add up individual scores
Technical	Interoperability	Does the application support FHIR APIs for patient data? No (0), Yes (1)	0 to 1	
Technical	Interoperability	Does the application have multiple form factors (e.g., phone, laptop, tablet)? No (0), Yes (1)	0 to 1	
Technical	Interoperability	Is the app available in iOS and Android formats? No (0), Yes (1)	0 to 1	
Technical	Performance	Does the application NOT crash close regularly?	0 to 2	Add up individual scores
Technical	Performance	Does the application have no perceptible delays in screen loading or log-in? No (0), Yes (1)	0 to 1	
Technical	Performance	Is there documentation the application content been updated within the past 3 months (2) or 6 months (1)? 3 months (2), 6 months (1), > 6 months (0)	0 to 2	

Clinical Domain

Domain	Title	Assessment Criteria	Points	Section Total
Clinical	Direct Evidence	Does the technology make a clinical claim to impact a health outcome? If yes, see below	0 = no and proceed to Credibility	Add up individual scores
Clinical	Direct Evidence	Does the technology provide health tips? If yes, see below	0 = no and proceed to Credibility	
Clinical	Direct Evidence	Is the clinical claim made by the technology supported by randomized control trial or meta-analysis?	0 = no; 4 = yes	
Clinical	Direct Evidence	Is the clinical claim made by the technology supported by non-randomized control trial or cohort study?	0 = no; 3 = yes	
Clinical	Direct Evidence	Is the clinical claim made by the technology supported by case-control studies; cross-sectional surveys; case reports?	0 = no; 2 = yes	

Clinical	Direct Evidence	Is the clinical claim made by the technology supported by expert opinion alone?	0 = no; 1 = yes	
Clinical	Direct Evidence	Is there any evidence to suggest clinical claim made by the technology is false or misleading?	0 = no; X = yes	
Clinical	Credibility	Produced by Recognized medical institution or organization	0 = no; 4 = yes	Add up individual scores
Clinical	Credibility	Produced by Recognized medical individual or team	0 = no; 3 = yes	
Clinical	Credibility	Endorsed by recognized medical institution/organization	0 = no; 2 = yes	
Clinical	Credibility	Endorsed by recognized medical individual/team	0 = no; 1 = yes	
Clinical	Credibility	None of the above	0 = yes	

Usability Doman (UX & UI)

Title	Performance Requirement	Points	Section Total
Visual Design & Readability	App should minimize need to scroll, and when scrolling is necessary users should be able to clearly identify when screens extend beyond the scroll line.	0 or 1	Add up individual scores
Visual Design & Readability	When possible, reduce the probability of data entry error by providing users with selectable options rather than requiring text entry.	0 or 1	
Visual Design & Readability	Apps should present information in easy to read format, e.g. chunked text vs. large paragraphs and appropriate text size	0 or 1	
Visual Design & Readability	Text should avoid use of jargon or acronyms that may not be familiar to users, particularly for lay users without clinical knowledge.	0 or 1	
App Navigation	Users should be able to easily identify where they are in the app and how to navigate to different destinations, including reversing actions. The navigational path should be logical, predictable, and easy to follow.	0 to 2	Add up individual scores
App Navigation	Navigating to primary tasks (from one area of the app to another) should require a minimal number of taps, swipes, or screens.	0 or 1	
App Navigation	The app’s main menu should be easily locatable and identifiable and labeled intuitively.	0 or 1	
App Navigation	On-boarding is completed in a timely manner and the user clearly understands the capabilities of the application after on-boarding	0 to 2	
Notifications, Alerts, Alarms	Users should be given the choice to opt out of automatic non-critical notifications and alerts.	0 to 2	Add up individual scores
Notifications, Alerts, Alarms	User's should be able to set reminders where app content is prescriptive for actions	0 = no; 1 = N/A; 2 = yes	

Help Resources & Support	Apps should have an easy-to-locate help section that consolidates all information intended to assist the user.	0 to 2	Add up individual scores
Help Resources & Support	Help features and informational links should be imbedded in the app when users may be likely to need them (?), with appropriate use of pop-ups or links.	0 to 2	
Help Resources & Support	Human support is provided in addition to digital support	0 or 1	
Utility	Single tasks should not require more than 1-2 screens and should minimize scrolling	0 to 2	Add up individual scores
Utility	Information and functions needed for a particular task or decision making (JTD) is grouped together in a single location	0 to 3	
Context & Personalization	Data appropriate for historical reference and trending should be stored and available to the user	0 or 1	Add up individual scores
Context & Personalization	Content and screens are personalized based on individual user situation and needs	0 to 2	
Context & Personalization	Must leverage data to add value to the user. 0 - Presentation only. 1 - Incorporates context to provide value from data. 2 - Predictive & AI usage of data	0 or 2	

Domain	Title	Assessment Criteria	Points	Section Total
Cost	Price	What is the initial purchase price? - Enter dollar amount<br>Free (2), listed free, but required a payment to access most features (1) and free trial, but need to pay to use after trial (0)	0 to 2	Add up individual scores
Cost	Price	Free with no Ads (1)<br>Free, but has Ads (0)	0 or 1	
Cost	Time	How much time does it take to set up? (2 mins or less (2); 3-5 mins (1); >5 mins (0))	0 to 2	Add up individual scores
Cost	Time	How much time does it take to understand the app navigation?<br>5 mins < (2); 6 mins > (1); > 7 mins (0)	0 to 2	
Cost	Time	Is there additional training required?<br>No (0), Yes (1)	0 or 1	

User requirements. User requirements outlined by pregnant women and midwives were collected from studies that reviewed or investigated user preferences for apps/digital health interventions [9,42,48]. Font in pink are the requirements outlined by pregnant women and font in blue are the requirements outlined by midwives.

Category	Focus Area	End User Type	Assessment Metric	Points (0 = none; 1 = partial; 2 = full)	Section Total
User Requirement	Education	Provider; pregnant woman/ midwives	The app must have educational content that is personalized and useful for me<br>• Information clutter/overload must be prevented	0 to 2	Total
User Requirement	Education	Provider; Patient/ family	The educational material and tracking in the app must help provide reassurance	0 to 2	
User Requirement	Education	Provider; Patient/ family	The educational material must be accurate and credible.	0 to 2	
User Requirement	Education	Provider; family	The app content should be presented in layman's terms/ easy to understand	0 to 2	
User Requirement	Social Support	Provider; Patient/ family	The app must connect to community resources that can aid me	0 to 2	Total
User Requirement	Social Support	Provider; Pregnant women	The app must allow me to share information with family/friends	0 to 2	
User Requirement	Social Support	Provider; Pregnant women	The app must facilitate/include connection w/ an online community of other pregnant women- forums, social media, direct chat	0 to 2	
User Requirement	Tracking /Logging	Provider; Pregnant women	The app must track medical details related to my pregnancy including; meds, appointments, other interventions	0 to 2	Total
User Requirement	Tracking /Logging	Provider; pregnant women, midwives	The app must have ability to connect/ message my healthcare team/ provider:<br>• without having a potential negative impact on patient-professional relationship<br>• without being time consuming	0 to 2	

User Requirement	Tracking /Logging	Provider; Pregnant women	The app must monitor changes in my body	0 to 2	
User Requirement	Tracking /Logging	Provider; Pregnant women	The app must monitor foetal development	0 to 2	
User Requirement	Tracking /Logging	Provider; Pregnant women	The app must store photos or videos of me (with or without including ultrasound images)	0 to 2	
User Requirement	Tracking /Logging	Provider; Patient/ family	The app must log my symptoms and view them historically<br>• Advice related to symptoms and suggestion for emergency consultation must be credible	0 to 2	
User Requirement	Prevention	Provider; Patient/ family	The app must provide personalized risk assessments	0 to 2	Total
User Requirement	Prevention	Provider; Patient/ family	The app must provide risk mitigation techniques	0 to 2	

App Behavior Change Scale (ABACUS) scoring: Behaviour change technique scale developed by [49] with examples.

Scale: item number and question		Definition	Example or further information	Points
**1. Knowledge and information**				
1.1	Does the app have the ability to customize and personalize some features?	Elements of the app can be personalized through specific tools or functions that are specific to the individual using the app.	• To select a disease type from among several available and then to follow a specific path or set of tools or systems.<br>• To select to receive emails or texts of a specific nature.<br>• To choose “yes” or “no” to a specific capability of the app would be considered personalization.	0 (no), 0.5 (partial) and 1 (full)

Scale: item number and question			Definition	Example or further information	Points
				• To create a personalized exercise plan.	
	1.2	Was the app created with expertise and/or Does the app provide information that is consistent with national guidelines?	This would be found in the about section or generally in the app.	• Does the app suggest 30 min of exercise each day?<br>• Does it recommend 5 veg and 3 fruit?<br>• Does it seek to build resilience and promote help seeking?<br>• Is there any evidence that the app was created by an expert? (doctor/professional body/university)	0 (no), 0.5 (partial) and 1 (full)
	1.3	Does the app ask for baseline information?	This includes BMI[a], weight, smoking rate, exercise, or drinking behaviors	• This might be at the set-up phase or in a profile setting.	
	1.4	Does the app provide instruction on how to perform the behavior?	The app is clear in telling the person how to perform a behavior or preparatory behaviors, either verbally, through video, or in written form.<br>NB: the behavior that is seeking to be changed, not information on how to use the app	• This could include showing person how to use gym equipment, sharing sample plans for action, instruction on suitable clothing, recipes, and general tips.	
	1.5	Does the app provide information about the consequences of continuing and/or	The app gives the user information about the consequences of behavior in general, this includes information about the relationship between the behavior and its	• Consequences may include health, feelings, or cost consequences.	

Scale: item number and question			Definition	Example or further information	Points
		discontinuing behavior?	possible or likely consequences in the general case. This information can be general or personalized.		
**2. Goals and planning**					
	2.1	Does the app ask for willingness for behavior change?	Is there a feature during setup where you describe how ready you are for behavior change?	• This may be in the form of a scale of readiness or in a question that asks the user to describe how ready you are.	0 (no), 0.5 (partial) and 1 (full)
	2.2	Does the app allow for the setting of goals?	The person is encouraged to make a behavioral resolution. The person is encouraged to set a general goal that can be achieved by behavioral means. This includes subgoals or preparatory behaviors and/or specific contexts in which the behavior will be performed. The behavior in this technique will be directly related to or be a necessary condition for the target behavior.	• This is the explicit noting of a goal or choosing a goal from one provided within the app.	
	2.3	Does the app have the ability to review goals, update, and change when necessary?	Involves a review or analysis of the extent to which previously set behavioral goals (regardless of short or long) were achieved.	• This is where a goal can be changed. This allows people to act on previously set goals and then revise or adjust where needed.	
**3. Feedback and monitoring**					

Scale: item number and question		Definition	Example or further information	Points
3.1	Does the app give the user the ability to quickly and easily understand the difference between current action and future goals?	Allows user to see how they are tracking against a goal and to see the difference between what they want to do and what they are currently doing. This will give some feedback on where they are at and what they need to change to get to where they want to be.	• This could be in the form of a graph or some other visual describing how close the user is to meeting their goals.	0 (no), 0.5 (partial) and 1 (full)
3.2	Does the app have the ability to allow the user to easily self-monitor behavior?	The app allows for a regular monitoring of the activity.	• Connects with watch that records daily steps that can be reviewed.<br>• Allows for easy logging of exercise or meditation?<br>• Allows for tracking of weight loss.<br>• Allows logging of daily alcoholic drinks or cigarettes.	
3.3	Does the app have the ability to share behaviors with others (including social media or forums) and/or allow for social comparison?	The app allows the person to share his or her behaviors on social media or in forums. This could also include a *buddy* system or a leaderboard.	• Share with Facebook or other socials<br>• Tell the user that they are doing x and at this time, other people like them are doing y	
3.4	Does the app have the ability to give the user feedback—either from a person or automatically?	The app is able to provide the person with feedback, comments, or data about their own recorded behavior. This might be automatic or could be personal.	• Does the app have a *coach* function?	

Scale: item number and question		Definition	Example or further information	Points
3.5	Does the app have the ability to export data from app?	The app allows for the export of information and progress to an external user.	• Export to a computer or to another user such as a doctor or fitness expert.<br>• Sharing to Facebook does not count.	0 (no), 0.5 (partial) and 1 (full)
3.6	Does the app provide a material or social reward or incentive?	App provides rewards for attempts at achieving a behavioral goal. This might include efforts made toward achieving the behavior or progress made in preparatory steps toward the behavior or in achieving a goal.	• Financial, either in returning money that was not spent on, for example, cigarettes or in paying someone to engage in a specific activity.<br>• Social or public, for example, congratulating the person for each day that he or she meets his or her exercise target.	
3.7	Does the app provide general encouragement ?	The app provides general encouragement and positive reinforcement on actions leading to the goal.	• This could include achievement badges or telling the user that they are a certain percentage closer to their goal.	
**4. Actions**				
4.1	Does the app have reminders and/or prompts or cues for activity?	The app prompts the user to engage in the activity. The app has the ability to give notifications or reminders to cue the behavior.	• This could be like the apple watch reminding you to stand or a meditation app telling you to meditate now.	0 (no), 0.5 (partial) and 1 (full)
4.2	Does the app encourage positive habit formation?	The app prompts explicit rehearsal and repetition of the behavior–not just tracking or logging.	• An example of this are the couch to 5 km apps that provide a training schedule.	

Scale: item number and question			Definition	Example or further information	Points
	4.3	Does the app allow or encourage for practice or rehearsal, in addition to daily activities?	App does not have a lock on activities or a number that you cannot exceed daily.	• This would include allowing the user to undertake extra activities in a single day.	0 (no), 0.5 (partial) and 1 (full)
	4.4	Does the app provide opportunity to plan for barriers?	The app encourages the person to think about potential barriers and identify ways of overcoming them.	• Alcohol app might give strategies for a night out that would normally be a big night.	
	4.5	Does the app assist with or suggest restructuring the physical or social environment?	The app prompts the person to alter the environment in ways so that it is more supportive of the target behavior.	• Might suggest locking up or throw away or their high-calorie snacks or take their running shoes to work.	
	4.6	Does the app assists with distraction or avoidance?	The app gives suggestions and advice on how the person can avoid situations or distract themselves when trying to reach their goal.	• For example, a smoking cessation app may suggest that the user not drink coffee if this is typically combined with smoking behaviors that they are trying to cease.	
