# Supplementary material for: Pregnancy Apps for Self-Monitoring: Scoping Review of the Most Popular Global Apps Available in Australia
Source: Int J Environ Res Public Health. 2023 Jan 5;20(2):1012. doi: 10.3390/ijerph20021012 (PMC9858738; doi:10.3390/ijerph20021012)
Supplement: Supplementary file 1 [file ijerph-20-01012-s001.zip › ijerph-2088281-supplementary/Supplementary_Material_appreview_4.01.23/Supplementary_TableS2_Statstables.pdf]

Supplementary material – stats tables

Table S2. Results of a Pearson correlation matrix to assess linear relationships between domain scores and global app rating of 31 apps was computed. Pearson *r*, *p* values and Confidence Intervals of *r* are reported.

Correlation matrix	Technical	Clinical	Usability	Cost and Time	End-user requirements	Global app ratings
**Technical**		*r* = 0.35 *p* = 0.06 CI = -0.008 to 0.63	*r* = 0.73 *p* = <0.0001 CI = 0.51 to 0.86	*r* = -0.25 *p* = 0.18 CI = -0.55 to 0.12	*r* = 0.68 *p* = <0.0001 CI = 0.42 to 0.83	*r* = 0.47 *p* = 0.01 CI = 0.13 to 0.70
**Clinical**	*r* = 0.35 *p* = 0.06 CI = -0.008 to 0.63		*r* = 0.36 *p* = 0.048 CI = 0.004 to 0.63	*r* = 0.08 *p* = 0.66 CI = -0.28 to 0.43	*r* = 0.61 *p* = <0.0001 CI = 0.33 to 0.79	*r* = 0.13 *p* = 0.49 CI = -0.24 to 0.46
**Usability**	*r* = 0.73 *p* = <0.0001 CI = 0.51 to 0.86	*r* = 0.36 *p* = 0.048 CI = 0.004 to 0.63		*r* = 0.12 *p* = 0.53 CI = -0.25 to 0.45	*r* = 0.65 *p* = <0.0001 CI = 0.38 to 0.82	*r* = 0.55 *p* = 0.001 CI = 0.25 to 0.76
**Cost and Time**	*r* = -0.25 *p* = 0.18 CI = -0.55 to 0.12	*r* = 0.08 *p* = 0.66 CI = -0.28 to 0.43	*r* = 0.12 *p* = 0.53 CI = -0.25 to 0.45		*r* = -0.08 *p* = 0.68 CI = -0.42 to 0.28	*r* = 0.12 *p* = 0.53 CI = -0.25 to 0.45
**End-user requirements**	*r* = 0.68 *p* = <0.0001 CI = 0.42 to 0.83	*r* = 0.61 *p* = <0.0001 CI = 0.33 to 0.79	*r* = 0.65 *p* = <0.0001 CI = 0.38 to 0.82	*r* = -0.08 *p* = 0.68 CI = -0.42 to 0.28		*r* = 0.36 *p* = 0.048 CI = 0.005 to 0.63
**Global app ratings**	*r* = 0.47 *p* = 0.01 CI = 0.13 to 0.70	*r* = 0.13 *p* = 0.49 CI = -0.24 to 0.46	*r* = 0.55 *p* = 0.001 CI = 0.25 to 0.76	*r* = 0.12 *p* = 0.53 CI = -0.25 to 0.45	*r* = 0.36 *p* = 0.048 CI = 0.005 to 0.63	

Pearson *r* = *r*, *p* = *p* values and CI = confidence interval. Purple denotes a significant positive correlation.
