# Supplementary material for: Pregnancy Apps for Self-Monitoring: Scoping Review of the Most Popular Global Apps Available in Australia
Source: Int J Environ Res Public Health. 2023 Jan 5;20(2):1012. doi: 10.3390/ijerph20021012 (PMC9858738; doi:10.3390/ijerph20021012)
Supplement: Supplementary file 1 [file ijerph-20-01012-s001.zip › ijerph-2088281-supplementary/Supplementary_Material_appreview_4.01.23/Supplementary_TableS3_Fullappscores.pdf]

Supplementary material – table of final app scores

Table S3. App scores for Individual Domains, Total Domain Score, Total BCT Score, Scorecard Score, Scorecard as ratings and Global app ratings. App rated from highest to lowest based on their Scorecard Score.

**App name**	**App code**	Technical (%)	Clinical (%)	UX/UI (%)	Cost/ Time (%)	User requirements (%)	**Total Domain Score (%)**	**Total BCT Score (%)**	**Scorecard Score (%)**	**Scorecard score as ratings**	**Global app ratings**
Baby2Body: Pregnancy Wellness	AR_22	62	50	84	73	42	62	63	63	3.11	4.7
Ovia Pregnancy Tracker	AR_41	77	25	96	73	69	68	49	59	2.90	4.9
Velmio: Pregnancy Tracker App	AR_73	56	0	75	43	38	43	55	49	2.50	4.9
Pregnancy Food Tracker ~Fittur	AR_86	52	13	42	33	24	33	60	46	2.30	4.9
Woman's Pregnancy	AR_14	35	63	55	70	51	55	35	45	2.24	4.6
WebMD Pregnancy	AR_74	53	63	61	63	57	60	28	44	2.20	4.5

GLOW Pregnancy & Baby Tracker (called Nurture on Apple)	AR_28	79	13	58	43	67	52	29	41	2.00	4.8
Pregnancy Tracker Week by Week	AR_58	60	13	63	53	26	43	35	39	1.95	4.7
Pregnancy + tracker app	AR_45	83	13	78	43	36	51	25	38	1.89	4.8
AMMA Pregnancy Tracker	AR_18	57	13	69	43	42	45	23	34	1.70	4.7
Sprout Pregnancy	AR_68	65	13	61	43	35	43	21	32	1.60	4.7
Stork - Pregnancy Tracker & Calendar App	AR_69	66	12.5	63	63	35	48	14	31	1.60	4.4
MomDiary: Week by week Pregnancy Tracker	AR_33	30	0	69	73	49	44	17	31	1.50	4.7

Pregnancy Companion	AR_48	36	0	59	73	28	40	22	31	1.50	4.5
Pregnancy tracker - Momly	AR_51	39	0	62	73	25	40	19	30	1.50	4.8
Pregnancy Tracker, Week by Week, Day by Day	AR_83	39	0	58	53	31	36	22	29	1.50	4.9
Pregnancy Week by Week	AR_62	42	0	55	83	27	42	14	28	1.40	4.2
Pregnancy Tracker + Countdown to Baby Due Date (Or BabyCentre on Apple)	AR_9	61	12.5	57	63	42	47	8	28	1.38	4.9
My Pregnancy	AR_37	37	0	66	100	13	43	11	27	1.35	4.8

Pregnancy App (pregnancy tracker on google play store)	AR_47	37	0	54	63	20	35	18	27	1.32	4.9
Pregnancy Tracker: Baby Bump	AR_84	21	0	42	100	16	36	8	22	1.00	4.6
Hello Belly: Pregnancy Tracker	AR_2	50	0	41	43	15	30	12	21	1.00	4.2
280days : Pregnancy Diary App	AR_17	39	0	52	33	29	31	11	21	1.00	4.8
myFetalLife	AR_38	34	0	34	43	26	28	13	20	1.00	4.4
Pregnancy Tracker	AR_56	36	0	48	53	22	32	6	19	0.94	4.3
Pregnancy Week by Week	AR_64	17	0	31	83	15	29	8	19	0.90	4.9
Pregnancy Tracker	AR_81	26	0	43	63	22	31	5	18	0.90	4.4
Babynote - Pregnancy Timeline	AR_23	13	13	28	70	20	29	4	16	0.82	4.4

Pregnancy calculator, symptoms, signs, calendar	AR_3	19	0	25	43	22	22	8	15	0.74	3.6
Happy Pregnancy Ticker	AR_29	5	0	43	43	10	205	9	15	0.70	4.2
Pregnancy Tracker Week By Week Pro Free	AR_59	17	0	37	53	17	25	3	14	0.70	4.2
**Average**		43.3	10.1	55.2	60.0	31.4	40.0	21.1			
